# A Case of Antisynthetase Syndrome in the Setting of SARS-Cov-2 Infection

**DOI:** 10.7759/cureus.40588

**Published:** 2023-06-18

**Authors:** Carlos Peña, Niketa Kalara, Pallavi Velagapudi

**Affiliations:** 1 Internal Medicine, Mount Sinai Medical Center, Miami Beach, USA

**Keywords:** mechanic hands, anti-jo-1 antibodies, anti-ej antibody, anti synthetase syndrome, inflammatory myositis

## Abstract

Antisynthetase syndrome is a complex autoimmune disorder, and one of the key criteria for diagnosis is the presence of myositis. Additionally, evidence of interstitial lung disease (ILD) is another important indicator for diagnosis; other clinical features associated with antisynthetase syndrome include arthritis, unexplained and persistent fever, Raynaud's phenomenon, and the presence of mechanic's hands. We report a case of a 36-year-old male who presented to the emergency department with shortness of breath and proximal muscle weakness in the setting of severe acute respiratory syndrome coronavirus 2 (SARS-Cov-2) infection, as his inflammatory markers were elevated and he exhibited features suspicious for antisynthetase syndrome, he was started on methylprednisolone 40 mg intravenously every eight hours, and a myositis panel was checked. In addition, a chest computed tomography (CT) exhibited ground-glass opacities which were compatible with coronavirus disease 2019 (COVID-19). A magnetic resonance image (MRI) of both thighs was done, revealing significant swelling and confirming the suspicion of myositis as his muscle strength in his lower extremities took significant time to improve. As days passed, his muscle strength improved significantly and his creatine phosphatase kinase (CPK) values trended down, indicating that his myositis was improving as well. He was transitioned to oral prednisone 60 mg daily and was discharged home with a rheumatology follow-up to define long-term treatment. A myositis panel revealed anti-glycyl-transferRNA synthetase (EJ) autoantibody positivity and a diagnosis was established. Our case revealed how sometimes laboratory values do not necessarily correlate with disease severity and how we have to do a thorough history of present illness and physical exam to think about unusual diagnoses before putting laboratory data into context.

## Introduction

Idiopathic inflammatory myopathies comprise a diverse group of rheumatic diseases with muscular and extra-muscular manifestations, including those affecting the skin, joints, and lungs, each with varying degrees of severity [1]. Antisynthetase syndrome is a rare autoimmune condition characterized by the presence of autoantibodies directed against aminoacyl transfer (t) RNA synthetases (aaRS), resulting in myositis, interstitial lung disease, Raynaud's phenomenon, fever, mechanic's hands, and arthritis [2]. The presence of these autoantibodies is a crucial diagnostic hallmark, and at least eight different antibodies targeting distinct aaRS have been identified: Jo-1 (antihistidyl tRNA synthetase), PL-7 (threonyl-tRNA synthetase), PL-12 (alanyl-tRNA synthetase), EJ (anti-glycyl-tRNA synthetase), OJ (isoleucyl-tRNA synthetase), KS (asparaginyl-tRNA synthetase), ZO (phenylalanyl-tRNA synthetase), HA (tyrosyl-tRNA synthetase); each defining a different disease phenotype [3].

Diagnosis of antisynthetase syndrome can be challenging due to its rarity and the multiple laboratory derangements observed, such as elevated creatine kinase (CK), the most sensitive indicator of inflammatory myopathy, and the elevation of both aspartate aminotransferase (AST) and alanine transaminase (ALT), which can be misinterpreted as underlying liver pathology instead of muscle disorder [4]. Interstitial lung disease is strongly associated with antisynthetase syndrome and is a major contributor to mortality [5].

We present a case of newly diagnosed antisynthetase syndrome in a patient with a concurrent severe acute respiratory syndrome coronavirus 2 (SARS-CoV-2) infection. The presence of the underlying pulmonary process made the diagnosis particularly challenging as even though his initial presentation was suspicious for myositis, at the time, the cause of his symptoms was not entirely clear as the serological findings didn't match the usual findings seen in this rare entity.

## Case presentation

A 36-year-old man with no significant past medical history presented to the Emergency Department with a chief complaint of proximal muscle weakness in both arms and legs that had been present for the last two months. Initially, he noticed difficulty reaching out for objects located overhead, but the symptoms progressed to the point where he had trouble getting up from a chair and climbing stairs, resulting in multiple mechanical falls. He also experienced difficulty chewing his food and noted tension in his neck. Additionally, he endorsed dryness and cracks on both of his hands that were not present before.

**Figure 1:**
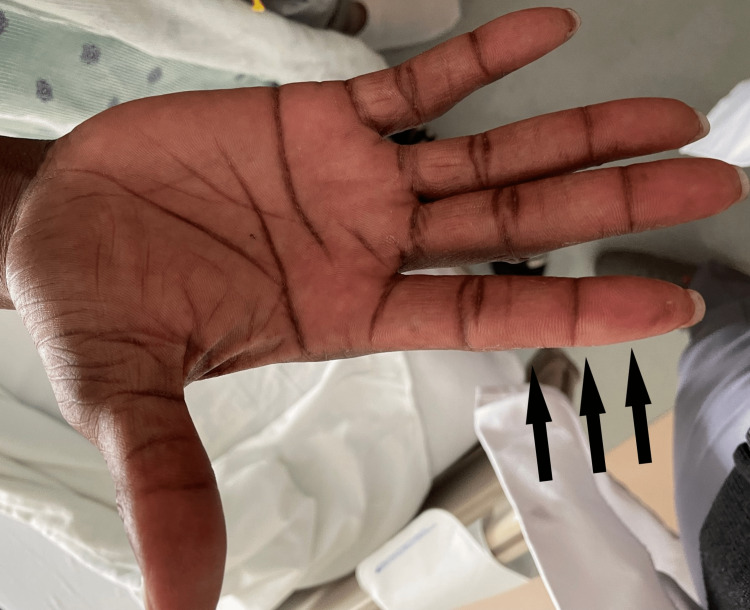
The patient's right hand. The skin was noted to be rough and crackled, also inter palmar lines were noted to be prominent. Dryness and scaling were more prominent on the radial aspect of the index finger.

In the days leading up to admission, he complained of fatigue, shortness of breath, and a sore throat. Upon initial evaluation, his physical exam was unremarkable except for muscle strength of 3/5 on arm abductors and 3/5 on hip flexors. No rashes were noted, but both hands showed rough, cracked skin predominantly on the radial aspect of both index fingers bilaterally (Figure 1).

Furthermore, bilateral crackles were auscultated bilaterally at the pulmonary bases. The patient disclosed a history of fever prior to the medical encounter, which was suspected to be attributable to a coronavirus disease 2019 (COVID-19) infection based on the presence of several positive sick contacts and the patient's unvaccinated status. Subsequently, a polymerase chain reaction (PCR) test for SARS-CoV-2 confirmed the patient's positive status for the virus.

On admission, the laboratory results (Table 1) showed multiple derangements. At this point, based solely on the physical exam and history, it was not possible to determine if all the symptoms endorsed by the patient were attributed to an inflammatory process of autoimmune etiology or if they were closely related to the underlying SARS-CoV-2 infection. Considering the possibility of inflammatory myositis, a decision was made to check a hepatic profile, acute phase reactants, lactate dehydrogenase (LDH), aldolase, and antinuclear antibody (ANA). It is noteworthy to mention that a JO-1 autoantibody was also checked, as this was the only item from the myositis panel that didn't require sending samples outside of our institution.

**Table 1:** Labs on admission

Labs On Admission	Result	Reference Range
White blood cell count (WBC)	7.5 10^3/uL	4.80 - 10.80 10^3/uL
Hemoglobin (Hb)	14.6 gr/dL	14.0 - 18.0 gr/dL
Mean corpuscular volume (MCV)	87.2 fL	79.0 - 92.2 fL
Platelets (PLT)	401 10^3/uL	150 - 450 10^3/uL
Sodium	134 mmol/L	136 - 145 mmol/L
Potassium	5.2 mmol/L	3.5 – 5.1 mmoL/L
Blood Urea Nitrogen (BUN)	7.29 mg/dL	7.0 - 18.0 mg/dL
Creatinine	1.63 mg/dL	0.70 - 1.30 mg/dL
Magnesium	2.3 mg/dL	1.6 - 2.6 mg/dL
Phosphorus	5.1 mg/L	2.5 - 4.9 mg/L
C-Reactive Protein	29.10 mg/L	0.00 - 3.00 mg/L
Erythrocyte sedimentation rate	22 mm/hr	0 - 20 mm/hr
Creatine Phosphokinase (CPK)	37,856 U/L	39 - 308 U/L
Aspartate Aminotrasferase (AST)	933 U/L	15.0 - 37.0 U/L
Alanine aminotransferase (ALT)	402 U/L	16.0 - 61.0 U/L
Lactated Dehydrogenase (LDH)	2154 U/L	84 - 246 U/L
Ferritin	1894 Ng/mL	26.0 - 388.0 Ng/mL
Aldolase	>150 U/L	< OR = 8.1 U/L
Anti-nuclear antibody	Positive. Titers 1/40	Negative
JO-1 antibody	Negative	Negative

**Figure 2:**
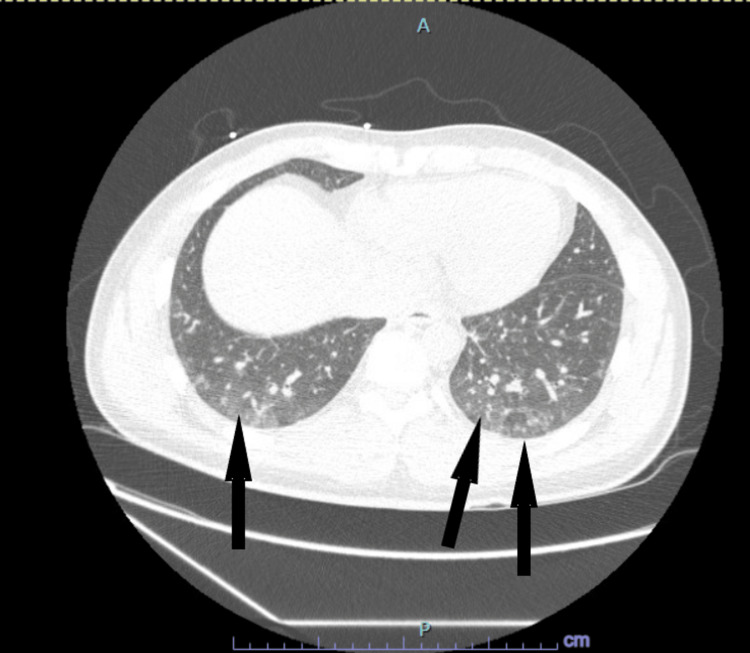
High-resolution chest CT scan showing hazy ground-glass opacities bilaterally on both posterior lung bases. Suspected to be related to viral pneumonia rather than ILD as ground glass opacities are predominantly peripheral and affect lower lobes whereas ILD might exhibit several abnormalities such as ground glass, reticular opacities, traction bronchiectasis, honeycombing, (and/or) non-emphysematous cysts, and typically affects at least 5% of a lung zone ILD: interstitial lung disease

In addition, a real-time polymerase chain reaction for SARS-CoV-2 was positive. Based on these results, rheumatology was consulted and evaluated the patient, ordering a myositis panel which included EJ, KU, MI-2, OJ, PL-12, PL-7, and SRP (signal recognition particle) autoantibodies. Additionally, a high-resolution CT scan of the chest was performed to rule out the presence of ILD (Figure 2). Although the respiratory symptoms the patient had endorsed were suspected to be related to SARS-CoV-2 infection, the other features described during the physical examination warranted further investigation to rule out the possibility of antisynthetase syndrome, which was among the list of differentials. The patient was started on 40 mg of intravenous methylprednisolone every eight hours with the aim of treating the inflammatory myopathy.

**Figure 3:**
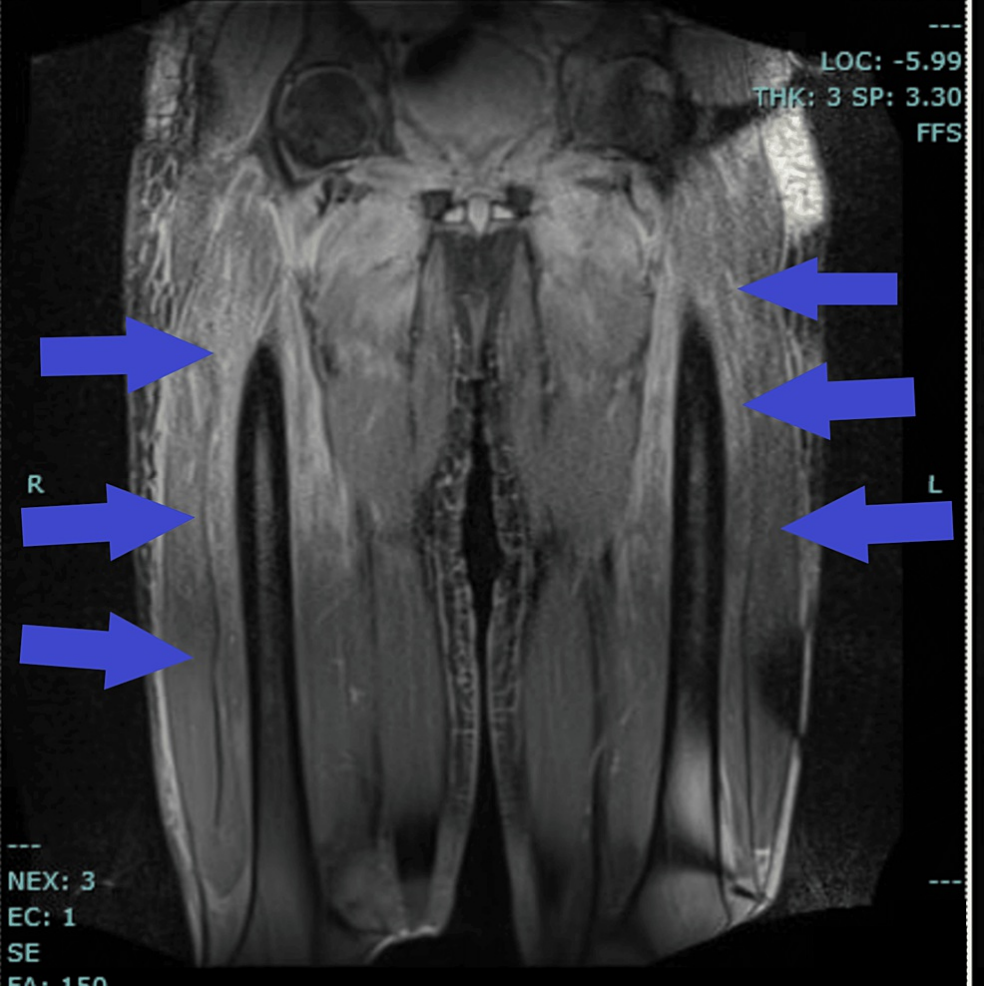
Bilateral MRI of the thighs. Diffuse bilateral intramuscular edema more prominent in the right hemi pelvis and right proximal thigh, findings that can be seen in inflammatory myositis

In the following days, his muscle strength improved significantly and his CPK started to trend down as he began showing improvement in his range of motion; from the initial value, it slowly trended down to 4921 U/L prior to discharge. As part of the workup, an MRI of both thighs (Figure 3) was indicated to aid in the diagnosis.

As the myositis panel was still pending, the decision was made to do an incisional biopsy of the thigh based on the findings seen on the MRI. Results revealed perivascular and endomysial lymphocyte infiltration, myofiber necrosis and regeneration, and peri-fascicular atrophy suggestive of dermatomyositis. At this time, we were able to decrease the dosage of methylprednisolone intravenously and switched to prednisone 60 mg by mouth. Since his symptoms were completely resolved, the decision to discharge the patient was made, and he was instructed on how to taper off the steroids prior to his follow-up visit with Rheumatology where he would have a long-term treatment prescribed for his condition.

Several days after discharge, the myositis panel results came in. Seven antibodies were checked and only the EJ antibody turned out positive, and a diagnosis was finally established.

## Discussion

Antisynthetase syndrome is a heterogeneous disorder that involves different organs and systems. Several diagnostic criteria have been proposed to guide the diagnosis, as it can be challenging [6]. Connors and Solomon have proposed criteria to facilitate the diagnosis of this rare entity (Table 2) [7,8]. However, the criteria established by Bohan and Peter in 1975, in an attempt to classify dermatomyositis and polymyositis, have fallen into disuse due to the heterogeneous spectrum of symptoms presented by this disease. Various attempts have been made to establish a standardized model that would accurately guide the workup for patients and improve the likelihood of establishing a prompt diagnosis, as some features tend to overlap between inflammatory myopathies. The most recent classification, established in 2017 by European Alliance of Associations for Rheumatology (EULAR)/American College of Rheumatology (ACR), intends to improve this situation; however, it does not include antisynthetase syndromes and immune-mediate necrotizing myopathy. In the case of antisynthetase syndrome, certain subsets of the inflammatory myositis disease spectrum can present with predominantly extra-muscular features rather than muscle weakness [9]. This illustrates the need for a high suspicion based on the history and symptoms reported by patients. In this particular case, the index of suspicion was high, as our individual exhibited clear signs of proximal weakness. However, this is not always the case, and it is important to keep in mind when approaching this type of case.

**Table 2:** Connors and Solomon criteria ILD: interstitial lung disease; ATS:

	Connors Criteria	Solomon Criteria
Serology	Presence of antibodies directed to aminoacyl tRNA synthetase	Presence of antibodies directed to aminoacyl tRNA synthetase
Clinical features	One or more of the following: evidence of myositis by Bohan and Peter criteria, evidence of ILD by American Thoracic Society (ATS) criteria, evidence of arthritis, persistent fever, Raynaud's phenomenon	Two major criteria or 1 major criterion plus 2 minor criteria. Major Criteria: ILD (Not explained by environmental, occupational, or drug exposures and not related to any other underlying disease) and evidence of myositis by Bohan and Peter criteria. Minor criteria: arthritis, mechanic's hands, Raynaud's phenomenon

When initially evaluating a patient who presents with muscle weakness, it is important to get a good history regarding the onset of the muscle weakness and associated symptoms. Also, when ordering different studies, it is important to keep in mind that among the different inflammatory myopathies, CK is one of the main muscle enzymes that will aid in diagnosis. However, the levels of CK will vary depending on the etiology, and these levels can help guide treatment and assess treatment response [10].

In this particular case, CK levels were significantly higher than what is expected to be seen in these disorders. Initially, it was thought that this patient was exhibiting symptoms suggestive of rhabdomyolysis, as he had muscle weakness, dark urine, and lab results revealed a CK level 10 times higher than the upper limit, along with acute kidney injury. Usually, the elevation of this enzyme correlates with the degree of muscle injury [11]. Our patient presented with levels unusually higher than the expected ranges seen in antisynthetase syndrome. Despite this fact, he fulfilled some of the other criteria for this pathology.

As mentioned before, antisynthetase syndrome has a broad spectrum of symptoms and clinical manifestations that have been associated with different autoantibodies. The disease presentation and prognosis will vary depending on the positivity of autoantibodies (Table 3) [1].

**Table 3:** Auto antibodies seen in antisynthetase syndrome, including disease spectrum and prevalence [1]

Antibody	Prevalence of Positivity	Associated Clinical Manifestations and Percentages
Anti-Jo-1	20-30%	Interstitial lung disease (70-90%), myositis (75-95%), arthritis (20-30%)
Anti-PL-7	5-10%	Interstitial lung disease (70-100%), myositis (70-80%)
Anti-PL-12	3-7%	Interstitial lung disease (40-60%), myositis (70-80%)
Anti-EJ	2-4%	Interstitial lung disease (60-80%), myositis (70-90%)
Anti-OJ	< 5%	Myositis (50-60%), interstitial lung disease (50-60%)
Anti-KS	< 5%	Myositis (40-60%), interstitial lung disease (40-50%)
Anti-Zo	Unknown	Myositis (20-30%), interstitial lung disease (10-20%)
Anti-Ha	Unknown	Myositis (20-30%), interstitial lung disease (10-20%)

A study observed that the PL-7 (+) and EJ (+) groups had a higher occurrence of ILD in comparison to the JO-1 (+) group. Additionally, the EJ (+) group displayed a greater likelihood of experiencing a rapid onset of dyspnea within four weeks of the onset of respiratory symptoms. However, the study found that the particular anti-ARS antibody positivity did not significantly affect the survival rate, implying that antisynthetase syndrome is a diverse condition and that the antibody specificity is only partially associated with the clinical course [12].

Previous studies, such as that of Watanabe et al., strongly suggest the association of EJ positivity with ILD [13]. However, recent evidence cautions against deciding on therapy based on serology, as this might result in unnecessary treatments despite having positive predictive values [14].

In terms of treatment, as ILD is the main predictor of mortality and prognosis, its presence or absence will significantly influence treatment. There are several alternatives available, but in general, initial treatment consists of steroids, and the dosing will depend on the severity of the disease, initially pulse steroid dosing such as methylprednisolone 1 gr IV for three days can be considered, followed by a readjustment on dosing as 1 mg/kg/day in the following days [5]. As a general principle, for maintenance therapy, these patients will need a steroid-sparing agent. Several agents, such as azathioprine and mycophenolate mofetil, have been suggested for patients with pulmonary involvement, with the use of tacrolimus and cyclosporine reserved for patients with a poor response. Additionally, the use of methotrexate has been described for patients with predominant myositis, showing a similar response to azathioprine. Moreover, the value of rituximab as a long-term agent for inflammatory myositis seems promising, as it has shown improvement in muscular symptomatology. Furthermore, the use of intravenous immunoglobulins (IVIG) has been described as well as a significant therapeutic agent as it has been shown to improve muscle strength; however, the impact on treating ILD appears to be limited [5,15].

Our patient responded considerably well to steroids, possibly achieving a stage where it would be possible to start long-term therapy.

## Conclusions

Our patient represents a challenging diagnosis as this disease is strongly associated with ILD. There was a high index of suspicion for inflammatory myositis as his symptoms were present for at least two months. As he also tested positive for SARS-Cov-2 and displayed shortness of breath, it was questioned at the moment of evaluation if he was exhibiting features compatible with ILD rather than the symptoms that can be seen within the spectrum of COVID-19 infection; the chest CT scan done earlier in his workup revealed that this was not the case. Moreover, a question that should be considered in this type of case is the relationship between SARS-CoV-2 and autoimmune diseases and its role in exacerbating some of the features of these diseases.

In addition, the labs revealed an abnormally high CK which is not typically observed in antisynthetase syndrome. Also, the most common autoantibody seen in this disease (JO-1) resulted negative, whereas EJ resulted positive; as has been mentioned before, this particular autoantibody is strongly associated with ILD, which our patient did not display. Our case shows that while the blood work suggested many differentials and possible managements for this individual, his presentation suggested different approaches and a broad array of options in terms of treatments, and also highlights the importance of a good physical exam and history taking when approaching difficult case presentations before defining therapeutic strategies.

## References

[1] Huang K, Aggarwal R (2020). Antisynthetase syndrome: a distinct disease spectrum. J Scleroderma Relat Disord.

[2] Galindo-Feria AS, Notarnicola A, Lundberg IE, Horuluoglu B (2022). Aminoacyl-tRNA synthetases: on anti-synthetase syndrome and beyond. Front Immunol.

[3] Mahler M, Miller FW, Fritzler MJ (2014). Idiopathic inflammatory myopathies and the anti-synthetase syndrome: a comprehensive review. Autoimmun Rev.

[4] Dalakas MC (2015). Inflammatory muscle diseases. N Engl J Med.

[5] Marco JL, Collins BF (2020). Clinical manifestations and treatment of antisynthetase syndrome. Best Pract Res Clin Rheumatol.

[6] Zanframundo G, Faghihi-Kashani S, Scirè CA (2022). Defining anti-synthetase syndrome: a systematic literature review. Clin Exp Rheumatol.

[7] Connors GR, Christopher-Stine L, Oddis CV, Danoff SK (2010). Interstitial lung disease associated with the idiopathic inflammatory myopathies: what progress has been made in the past 35 years?. Chest.

[8] Solomon J, Swigris JJ, Brown KK (2011). Myositis-related interstitial lung disease and antisynthetase syndrome. J Bras Pneumol.

[9] Leclair V, Lundberg IE (2018). New myositis classification criteria-what we have learned since Bohan and Peter. Curr Rheumatol Rep.

[10] Ashton C, Paramalingam S, Stevenson B, Brusch A, Needham M (2021). Idiopathic inflammatory myopathies: a review. Intern Med J.

[11] Gupta A, Thorson P, Penmatsa KR, Gupta P (2021). Rhabdomyolysis: revisited. Ulster Med J.

[12] Zhan X, Yan W, Wang Y, Li Q, Shi X, Gao Y, Ye Q (2021). Clinical features of anti-synthetase syndrome associated interstitial lung disease: a retrospective cohort in China. BMC Pulm Med.

[13] Watanabe K, Handa T, Tanizawa K (2011). Detection of antisynthetase syndrome in patients with idiopathic interstitial pneumonias. Respir Med.

[14] Basuita M, Fidler LM (2022). Myositis antibodies and interstitial lung disease. J Appl Lab Med.

[15] Pipitone N, Salvarani C (2020). Up-to-date treatment and management of myositis. Curr Opin Rheumatol.

